# Health literacy in Indigenous people with chronic disease living in remote Australia

**DOI:** 10.1186/s12913-019-4335-3

**Published:** 2019-07-26

**Authors:** Haunnah Rheault, Fiona Coyer, Lee Jones, Ann Bonner

**Affiliations:** 1 School of Nursing, Queensland University of Technology, Brisbane, Australia; 2 Advanced Heart Failure and Transplant Unit, The Prince Charles Hospital, Rode Road, Chermside, Brisbane, QLD 4032 Australia; 3 Intensive Care Services, Royal Brisbane and Women’s Hospital, Brisbane, Australia; 4 Institute of Health and Biomedical Innovation, Queensland University of Technology, Brisbane, Australia; 5 Kidney Health Service, Metro North Hospital and Health Service, Brisbane, Australia; 6 NHMRC Chronic Kidney Disease Centre of Research Excellence, University of Queensland, Brisbane, Australia

**Keywords:** Health literacy, Health literacy questionnaire, HLQ, ATSI health, Chronic disease, Rural and remote health

## Abstract

**Background:**

Health literacy is strongly associated with health outcomes and is important for health policy and service delivery. Low health literacy was reported in 59% of Australian adults, however, there is no national data on the health literacy of Aboriginal and Torres Strait Islander (ATSI) peoples. The ATSI population in Australia experience a notable gap in health outcomes compared with non-Indigenous Australians which is due, in part to a higher prevalence of chronic diseases. The health outcome gap is more pronounced in rural and remote locations. This study aims to establish the health literacy profile of ATSI adults with chronic disease living in remote North-West Queensland Australia, and to investigate associations between the Health Literacy Questionnaire (HLQ) domains and self-reported chronic disease and demographic characteristics.

**Methods:**

Using a cross-sectional design, 200 ATSI adults with a diagnosis of chronic disease/s (cardiovascular disease, diabetes, respiratory disease and/or chronic kidney disease) were recruited from two sites with the assistance of Aboriginal Health Workers. Data were collected using the HLQ, a multidimensional 44 item instrument to assess nine domains of health literacy. Demographic and health data were also collected. Analysis of variance using backwards modelling was used to determine predictors of health literacy.

**Results:**

Participants were mostly male (53.5%) and aged between 19 and 89 years. The most prevalent chronic disease was cardiovascular disease (74%) followed by diabetes (67.5%). More than half (62%) had two or more chronic diseases. There was at least one independent predicator for each of the nine health literacy domains. Age, number of chronic diseases, gender, and level of education were all highly significant predictors of health literacy.

**Conclusion:**

Improved health literacy will enable individuals to take an active role in their health. Understanding the health literacy of ATSI adults is a crucial first step. Our findings can assist Australian healthcare organisations to review their health literacy responsiveness and examine ways to improve patients’ needs and health capabilities to better support people to engage in effective self-management for chronic diseases.

**Electronic supplementary material:**

The online version of this article (10.1186/s12913-019-4335-3) contains supplementary material, which is available to authorized users.

## Background

Health literacy is not a new concept and is increasingly being recognised as a powerful and important factor in the delivery of healthcare which not only includes the consumer, but the healthcare system at large. Health literacy remains one of the least understood and neglected factors [1] despite adequate health literacy being a significant contributor to improved health outcomes [2]. Health literacy far surpasses the ability to read and write and encompasses a wider array of competencies to manage one’s health. Health literacy involves the consultation, engagement and communication with healthcare providers and the journey of navigation through complex healthcare systems [1–4]. Health literacy also encompasses the critical appraisal of health information from different sources, the social support needed to access services and maintain good health, and understanding ones’ rights as healthcare consumers [1–4]. People with lower health literacy have less knowledge of their health problems [5, 6], less knowledge on how to effectively self- manage [7], have lower uptake of health screenings [8], lower rates of engagement in health promoting behaviours [9], lower medication adherence [10], higher rates of hospitalisation [11, 12], experience 30-day hospital readmission after discharge [13], and have a poorer overall health status [9].

Australia is a vast and diverse nation of 24 million people [14] with approximately one third of the Australian population living outside major cities [15]. Aboriginal and Torres Strait Islander (ATSI) people are the First Peoples of Australia inhabiting the Australian continent for over 60,000 years and they currently comprise about 2.8% of the Australian population [14]. The ATSI peoples, also referred to as Indigenous peoples, are not one group of people, but hundreds of discrete groups. The groups are connected through complex kinship systems and social structures [16], each conversing in their own distinct languages passing down the cultural and social traditions via performance, drawings, protection of important sites and storytelling [17]. Traditionally nomadic or semi-nomadic hunter-gathers [18], ATSI people now predominately live in urban centres of Australia with 80% living in urban areas and 20% living in remote areas of Australia [15].

Cross-country comparisons of Australia, New Zealand, North America and Canada show that life expectancy is substantially lower for Indigenous peoples [19, 20]. In Australia, ATSI people experience widespread socioeconomic disadvantage and health inequity [14, 21, 22]. ATSI people are a younger population than the non-Indigenous Australian population [23]. In 2016, more than half (53%) ATSI people were aged less than 25 years compared with 13% of non-Indigenous population [23]. In direct contrast, the proportion of ATSI people aged 65 years or older was substantially smaller (4.8%) compared with 16% for non-Indigenous Australians [24]. The current life expectancy is estimated to be 10.6 years less for ATSI men and 9.5 years for ATSI women (69.1 years for men and 73.7 years for women) compared with the non-Indigenous population [25]. The life expectancy for ATSI males living in remote areas of Australia is 0.7 years lower than their counterparts residing in major cities (67.3 years compared with 68.0 years). Similarly for ATSI women living in remote areas of Australia, the life expectancy is 0.8 years lower than those living in the major cities (72.3 compared with 73.1 years) [26]. The differences may be due to the high burden of chronic disease in remote areas of Australia along with social, educational and other determinants of health.

Chronic disease is a global health concern, and in Australia it is the leading cause of morbidity and death [27]. The burden of chronic disease is far greater for ATSI people [28]. Aboriginal and Torres Strait Islander people have five times the rates of diabetes and four times the rate of chronic kidney disease than non-Indigenous Australians [29]. Two-thirds of ATSI people reported having one chronic disease, and one third reported having three or more chronic diseases [28], thus a current key focus of the Australian health system is therefore the prevention and improved management of chronic disease in the ATSI population [27]. Chronic disease management is both challenging and complex for individuals and the healthcare system at large requiring a wide range of health literacy skills and support from social networks [30, 31]. Recognising that ATSI people experience high rates of chronic disease has been the first step, the current challenge is to anticipate chronic disease comorbidity and to invest in promoting self-management and disease specific education through improving health literacy to extend both the quality and duration of ATSI peoples’ lives. Examining health literacy in a community can benefit improvement and re-evaluation in clinical service delivery, public health education, policy development and both community and individual participation in health [2, 32].

In 2006 health literacy was assessed in Australians (the only national survey) with 59% having low levels of health literacy [33]. This survey excluded remote areas of Australia and ATSI status was not recorded [33]. There is no national data on the health literacy levels of ATSI peoples. However, health disparity- a higher risk of disease and disability, can be attributed to poor health literacy skills [34]. There has been limited studies exploring health literacy in ATSI people. Three studies used qualitative methods to: explore ways at improving health education and communication [35] the individual, social and cultural aspects of health literacy relative to cancer [36]; and a study protocol to examine the effect of a medication education program on the health literacy of Indigenous Australians [37]. Two studies have measured the functional health literacy of ATSI people. Parker and Jamieson (2010) used the Rapid Estimate of Adult Literacy in Dentistry to measure functional dental health literacy [38]. Lakhan and colleagues (2010) assessed functional health literacy as well as communication of ATSI peoples attending a primary health care clinic [39]. However, no studies have assessed the multidimensional aspects of health literacy in ATSI peoples with chronic disease.

### Aim

The aim of this study was to establish the multidimensional health literacy profile of ATSI adults with chronic disease living in remote North-West Queensland. The second aim was to investigate associations between the independent Health Literacy Questionnaire (HLQ) domains, and self-reported chronic disease and demographic characteristics.

## Methods

### Study design

This study used a cross-sectional survey design.

### Setting

**Fig. 1:**
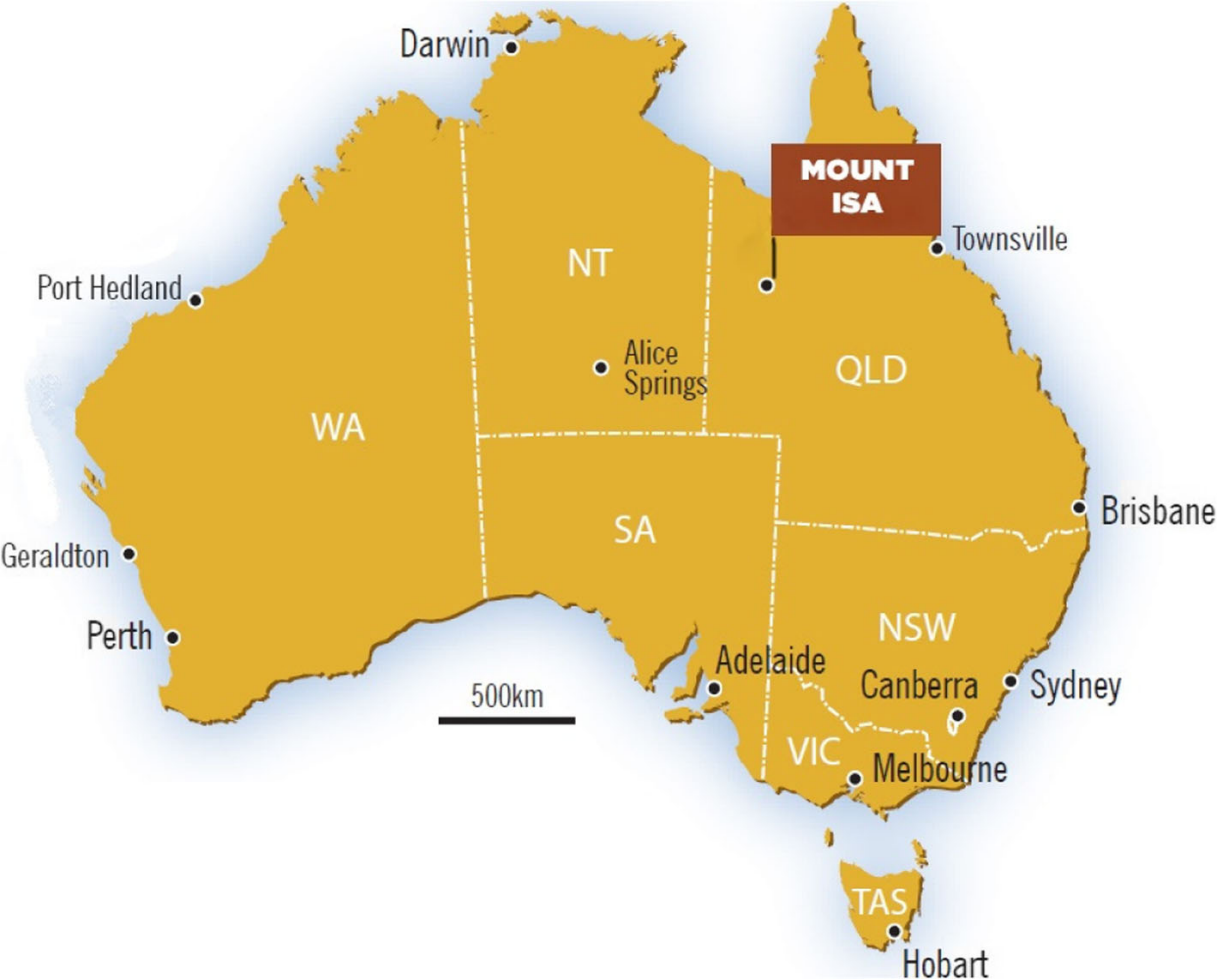
Map of Australia. Map of Australia highlighting the city of Mount Isa in comparison to the major capital cities of Australia. Permission has been obtained to use and adapt the image from the following source: www.isarodeo.com.au/plan-your-trip/the-facts/map-australiamountisa/ [67]

Over one quarter (28.7%) of the Indigenous population of Australia live in Queensland with 15.5% living in remote areas and large proportion (39.4%) living in very remote areas [15]. The study was conducted in the remote north-west city of Mount Isa, Queensland, Australia, approximately 1,800 km from Brisbane (the state capital city) (see Fig. 1). Mount Isa is a mining town with a population of approximately 22,000 people, 25% of whom identify as either Aboriginal and/or Torres Strait Islander [40]; equating to approximately 5,500 people.

Participants were recruited from chronic disease specialty outpatient clinics from both Mount Isa Hospital and The Prince Charles Hospital. At Mount Isa hospital there are specialty outpatient clinics for various chronic diseases each week (1 cardiology, 2 diabetes, 1 respiratory and 1 chronic kidney disease). These clinics are operated by either specialist nurse or by a visiting (fly-in fly-out) medical specialist. Aboriginal Health Workers work alongside nurses and medical staff in these clinics. The Prince Charles Hospital, located in Brisbane, serves as a referring specialist tertiary cardiac service for ATSI people residing in Mount Isa. Patients who reside in Mount Isa and attending outpatient cardiology clinics (2 clinics per week), a medical specialist led clinic, were also recruited into the study.

### Sampling and sample size

#### Sampling procedure

Crucial to conducting research within ATSI populations, early and respectful discussions must occur with the Elders of the community. For this study, Kalkadoon Elders (Indigenous peoples of Mount Isa) and the Kalkadoon Native Title Aboriginal Corporation were consulted whom provided their endorsement of the study. Also, two trained Aboriginal Health Workers (1 male, 1 female) from Mount Isa Hospital assisted in refining the study procedures and then acted as research assistants. One Aboriginal Health Worker (male) was engaged as the Cultural Advisor for this study, ensuring cultural safety and to act as a cultural broker between the research team and the community.

Using convenience sampling, potential participants were approached by either trained Aboriginal Health Workers (1 male, 1 female) or the First Author and were verbally informed of the study aims. A written (English) participant information sheet was provided and real aloud. Participants were offered a choice of gender of Aboriginal Health Worker to ensure cultural and sensitivity protocols were respected. Participation was on a voluntary basis and verbal consent was obtained. Participants were informed of their right to withdraw at any time without affecting their care or future access to health care. Both the HLQ and a self-reported demographic and health data questionnaire were read aloud verbatim to every participant (in English) taking 20–30 min (total) to complete. Questionnaires were completed in private outpatient clinic rooms, or in a place of the participant’s choice (a respectful way to engage ATSI participants). This included but was not limited to a participant’s residence, a local park, or within a local community health centre. Inclusion criteria were adults ≥18 years, self-identified as Aboriginal and/or Torres Strait Islander, and medically diagnosed with one or more chronic disease/s including cardiovascular disease, diabetes, respiratory disease and/or chronic kidney disease. Exclusion criteria were those receiving healthcare from the first author (deidentified for review), medically diagnosed with cognitive impairment or severe mental illness. A response rate was not captured. Data collection took place between February and November 2017.

#### Sample size

This study involves ATSI adults in remote Australia. Representative surveys for these populations are difficult due to complex methodology, and reliable population health data for these populations are not available [41]. The standard deviation is unknown in this remote population, as such the sample calculation was powered using a rule-of-thumb method to allow for multivariable modelling. The projected sample size was 220 participants, calculated by applying the subjects-to-variables ratio, based on 5–10 participants multiplied by the number of variables [42].

#### Instruments

The HLQ is a multidimensional 44-item questionnaire measuring nine independent domains of health literacy (see Table 1) [4]. It captures a profile of an individual’s health literacy abilities across the functional, communicative, social and critical dimensions [43]. The nine domains, comprehensively described by Osborne et al., (2013) measure the capabilities “of an individual to understand, engage with, and use health information and health services” [4]. The additional value in using the HLQ is that it also “reflect the capabilities of an organisation to provide services that enable a person to understand, engage and use their health information or services” [4]. Health literacy domains 1–5 are scored using a 4-point scale from ‘strongly disagree’ to ‘strongly agree’, and domains 6–9 have a 5-point scale recording self-reported capabilities from ‘cannot do or always difficult’ to ‘always easy’ [4]. This instrument does not have one total health literacy score, instead there is a score for each of the nine health literacy domains providing an indication of the strengths and limitations of the respondent [32]. A low score indicates that the respondent has difficulties within the domain, and a high score indicates greater health literacy ability. The psychometric properties of the HLQ prove to be highly robust [44]. Bayesian confirmatory factor analysis has confirmed high composite reliability in all 9 HLQ domains (Cronbach’s alpha ≥0.8) [44]. The HLQ is endorsed by the WHO [45] and has been translated into multiple languages and used to examine health literacy across many different populations, cultures and settings [3, 10, 46–52] including Australia [31, 45, 48, 53–56].

**Table 1:** Health Literacy Questionnaire health literacy domains

Health literacy domains	No of items
Healthcare provider support	4
Having sufficient health information	4
Actively managing health	5
Social support for health	5
Critical appraisal	5
Active engagement with healthcare providers	5
Navigating the healthcare system	5
Ability to find good health information	5
Reading and understanding health information	5

A 10 question self-reported demographic and health data questionnaire followed the HLQ (see Additional file 1). Both Aboriginal Health Workers assisted with the development of the demographic and health data questionnaire seeking data about gender, age, income, education, living arrangements, chronic disease health history, use of local medical services, and current medication use. Collaboration with Aboriginal Health Workers was focused on cultural appropriateness and sensitivity issues for the wording and structure of the demographic and health data questionnaire.

### Statistical analyses

Descriptive statistics and HLQ scale scores were analysed used using SPSS® version 23 [57] including measures of central tendency (means, medians) and dispersion (standard deviations and ranges) to describe the total sample. Analysis of variance (ANOVA) was used for analysis of the nine HLQ domains and self-reported variables including attendance at the local Aboriginal community controlled medical centre (AMC), gender, income, age, education and number of diagnosed chronic diseases, with post hoc testing where applicable. Effect size (ES) were calculated using Cohen’s d, and interpretation of ES was adopted from Cohen [58]; a small ES score = 0.2–0.5, medium ES = 0.5–0.80 and large ES > 0.8. Multivariable models were created using backwards modelling to identify key variables associated with health literacy. The variables were gender (male, female), age (< 55, > 55; determined by sample median), household income (<$30,000, $30,000–$40,000, ≥$40,000, prefer not to answer), attendance to a medical appointment at the local AMC in the past 4 weeks (yes, no), education levels (primary school, secondary school, Technical and Further Education [TAFE]/University/Trade qualification, prefer not to answer) and number of diagnosed chronic diseases (1, ≥2). The significance was set at a *p* < 0.05 and 95% confidence intervals (CI) were calculated where appropriate. Residuals were looked at for normality, and homogeneity of variance were within reasonable limits.

## Results

### Demographic and health characteristics

A total of 200 people (53.5% males) ranging from 19 to 89 years (mean = 55, SD = 15.7) participated. A majority reported having cardiovascular disease (74%), 67.5% had diabetes, 26.5% respiratory disease and 24.5% chronic kidney disease. More than half (62%) had two or more chronic diseases. The highest level of education was secondary school (41%), followed by primary school education (19.5%). A high proportion (71%) of participants attended a medical appointment at the local AMC within the preceding 4 weeks. Table 2 presents the demographic and health characteristics.

**Table 2:** Demographic and health characteristics

	Total *n* = 200	Men *n* = 107	Women *n* = 93
Age (years), Mean (SD)			
*Range 19–89*	54.6 (15.7)	55 (16)	54.1 (16.3)
Gender, n (%)		107 (53.5)	93 (46.5)
Education, n (%)			
Primary	39 (19.5)	26 (24)	13 (14)
Secondary	82 (41)	41 (38)	41 (44)
TAFE/University/Trade qualification	24 (12)	9 (8)	15 (16)
Prefer not to answer	55 (27.5)	31 (29)	24 (26)
Annual household income, n (%)			
< $30,000	34 (17)	15 (14)	19 (20)
$30,000–$40,000	60 (30)	36 (34)	24 (26)
> $40,000	54 (27)	24 (22)	30 (32)
Prefer not to answer	52 (26)	32 (30)	20 (22)
Number people living in household, Mean (SD)			
*Range 1–12*	4.79 (2.28)		
Chronic disease profile, n (%)			
Cardiovascular disease	148 (74)	82 (77)	66 (71)
Diabetes	135 (67.5)	70 (65)	65 (70)
Respiratory disease	53 (26.5)	26 (24)	27 (29)
Chronic kidney disease	49 (24.5)	31 (29)	18 (19)
Other reported chronic disease	4 (2)	2 (2)	2 (2)
1 chronic disease	76 (38)	43 (40)	33 (35.5)
≥ 2 chronic diseases	124 (62)	64 (60)	60 (64.5)
Number of medications taken per day			
Median, (IQR)	3 (2–5)		
*Range 1–20*			
Outpatient clinic review past 4 weeks, n (%)			
Attended local Aboriginal community controlled medical centre in Mount Isa	142 (71)	80 (75)	62 (67)
Attended a specialist medical clinic at Mount Isa Hospital	81 (40.5)	48 (45)	33 (35)
Attended a specialist nurse-led clinic at Mount Isa Hospital	83 (41.5)	45 (42)	38 (40)

Abbreviations: *n* = number, *SD* = Standard deviation, *IQR* = Interquartile range, *TAFE* = Technical and Further Education

### Health literacy questionnaire

Total mean scores for each HLQ domain are displayed in Table 3. Due the different scoring of the HLQ, of the first 5 health literacy domains with a scoring range from 1 (lowest: strongly disagree) to 4 (highest: strongly agree), domain 4 *Social support for health* had the highest mean score (mean = 2.84, SD 0.52) and lowest was seen in domain 5 *Critical appraisal* (mean = 2.41, SD 0.55) which was the lowest mean score across all 9 HLQ domains. All 5 domains had an overall mean score of < 3, which suggests that participants are somewhat ambivalent on their feelings of being supported by healthcare providers, having sufficient health information, ability to actively managing their health, having enough social support for their health and their ability to critically appraise health information. Within the HLQ domains 6 to 9 the scoring range is from 1 (lowest: cannot do/always difficult) to 5 (highest: always easy to do), and gauges how difficult or easy tasks are for respondents. The highest mean score was in domain 6 *Active engagement with healthcare providers* (mean = 3.14, SD 0.72) which was also the highest mean score across all 9 HLQ domains. The lowest was seen in domain 9 *Reading and understanding health information* (mean = 2.82, SD 0.78). Domain 8 *Ability to find good health information* (mean = 2.89, SD 0.73) and domain 9 *Reading and understanding health information* were difficult for participants to accomplish.

**Table 3:** Health Literacy Questionnaire scores (n = 200)

HLQ domain	Mean (SD) [95% CI]
Range 1 (lowest) – 4 (highest)	
1. Healthcare provider support	(0.52) [2.69, 2.83]
2. Having sufficient health information	(0.52) [2.51, 2.66]
3. Actively managing health	(0.49) [2.57, 2.70]
4. Social support for health	(0.52) [2.76, 2.91]
5. Critical appraisal	2.41 (0.55) [2.33, 2.49]
Range 1 (lowest) – 5 (highest)	
6. Active engagement with healthcare providers	(0.72) [3.01, 3.24]
7. Navigating the healthcare system	(0.75) [3.01, 3.23]
8. Ability to find good health information	(0.73) [2.79, 2.99]
9. Reading and understanding health information	2.82 (0.78) [2.72, 2.93]

Abbreviations: *HLQ* = Health Literacy Questionnaire, *SD* = Standard deviation, *CI* = Confidence interval

### Predictors of health literacy

Results showed that age, gender, number of chronic diseases, education levels and income were associated with health literacy. Attending a medical appointment at the local AMC in the preceding 4 weeks was not significant. Characteristics of higher health literacy included being < 55 years of age, female, having only one chronic disease, higher levels of education and an income of <$30,000 (see Additional file 2). Bivariate analyses of the six predictor variables and ES for each HLQ domains (see Additional file 3). At a bivariate level mostly small to medium ESs were found across the nine HLQ domains, with the exception of the variable attending a medical appointment at the local AMC in the preceding four weeks which had a very small ES across all nine HLQ domains. There was at least one independent predictor for each of the nine health literacy domains. Age (< 55 years) had higher health literacy mean scores across 8 of the 9 domains, followed by having 1 chronic disease (5 of the 9 domains), being female (3 of the 9 domains), having higher levels of education (2 of the 9 domains) and lower income earners (1 of the 9 domains). Four HLQ domains had 3 associations for higher health literacy (domains 2, 5, 7, 9) and 3 domains had 2 variables related with higher health literacy (domains 6, 8). We found 3 similar variables (< 55 years, female, 1 chronic disease) significantly predicted 3 HLQ domains being; *Critical appraisal*, *Navigating the healthcare system,* and *Reading and understanding health information*. Variables including age (< 55 years), income (<$30,000) and having a higher level of education level were associated with domain *Having sufficient health information*. Age (< 55 years) predicted *Actively managing health* and *Social Support*, whilst higher levels of education was associated with HLQ domain *Healthcare provider support.*

## Discussion

Our study was the first to describe health literacy in the ATSI population who have at least one chronic disease. We found patterns in predictors for higher health literacy levels for which health providers can use to improve health literacy responsiveness. We found that age and the number of chronic diseases were major contributors to health literacy abilities. Being less than 55 years of age was strongly associated with higher levels of health literacy across almost all domains. Potentially, younger adults have had more opportunity for further education which has been associated with less chronic disease in the Australian population [28]. The age-related predictors also occurred across 5 similar HLQ domains if the person had one chronic disease, and if the person was female (seen amongst 3 similar domains). Having only one chronic disease was a predictor of higher health literacy across 5 HLQ domains. Clearly increase in age or having comorbid chronic diseases made it more challenging for this population to navigate the healthcare system, and to find and appraise health information. Approximately one quarter of the Australian population have two or more chronic conditions [59] which means that more complex self-management is required, and that can impose a significant burden on individuals and their families [60]. Having only one chronic disease is likely to be easier to manage daily treatment regimens as opposed to having 2 or more chronic diseases; number of chronic diseases reinforcing the notion that health literacy is contextual [30].

Higher levels of education predicted health literacy domains *Healthcare provider support* and *Having sufficient health information*. It could be that having more education could enable the person to feel more confident with communicating with healthcare providers and this could increase the perception of feeling understood and supported. However, higher levels of education was not associated with abilities requiring advanced cognitive skills to critically analyse information to exert greater control over life events and situations. Both low (<$30,000) and high income (>$40,000) were associated with the domain *Having sufficient health information*. However about 25% of the participants preferred not to state their income precluding an understanding of the associations between health literacy and income in this study. Further research on this association in this population is needed.

Tradition models of healthcare delivery separate services into disease-related clinical silos whereby people attend numerous health-related appointments, often in different locations and/or on different days. From a health literacy perspective, integrated models of chronic disease comorbidity may reduce confusing and/or conflicting information provided. Changes in service delivery models that are person-centred rather than disease-centred could lead to improved functional, communicative and critical health literacy abilities and potentially could reduce the contextual nature of health literacy. The constant interactive process between consumers, communities, healthcare providers and healthcare organisations, [2] and through re-examining the health literacy responsiveness of organisations and the training of staff in health literacy principles, may facilitate improved health literacy in this population. Efforts need to be put into reviewing health information, including mode of delivery, by whom and when this information is delivered to this population with chronic disease/s.

Interpretation of the overall HLQ mean data suggest that participants felt able to actively engage with healthcare providers (domain 6) and navigate their way through the healthcare system (domain 7). This was not entirely surprising as Mount Isa is a small city with only one hospital which employs Aboriginal Health Care Workers and Aboriginal Liaison Officers who work across inpatient and outpatient services who are able to assist with navigation and facilitate both the engagement and communication with healthcare providers. There is also a specific AMC (which also employs Aboriginal Health Care Workers) in Mount Isa and only a few General Practices, so it could be that due to a lack of choice, coupled with the support offered by Aboriginal Health Care Workers, that this population does feel relatively able to actively engage with healthcare providers and navigate their way through the healthcare system. Despite this, participants overall found reading and understanding health information, being able to find good health information, and to critically appraise health information difficult to do. These difficulties were also found in the domains of having sufficient health information and being able to actively manage their health. If individuals cannot find health information or appraise the usefulness of that information, then being able to adhere with self-management activities are likely difficult to do.

Despite a large proportion of participants (71%) attending a medical appointment at the local AMC within the preceding four-weeks, there was no association with higher levels of health literacy in any HLQ domain. We found this interesting, as this clinic provides close and extensive follow-up care (including emotional and social support from Aboriginal Health Workers) of patients, and assistance with medications (cost and delivery) through government initiative scheme, “Closing the Gap”. We do not suggest that simply attending one appointment at the AMC would suddenly make one health literate. Our cohort had 62% of individuals with ≥2 chronic diseases, and it is unlikely that this one clinical interaction was their first visit/interaction at the AMC. Surprising, this variable was not significant with HLQ domains of *Healthcare provider support, Social support for health* or *Active engagement with healthcare providers*. It could be inferred that recruitment and retention of clinicians to remote and isolated locations and to work in an AMC is challenging, and that might inhibit the formation of trusting relationships between clinicians and patients.

In this study, we were surprised that we did not find high levels in *Social support for health* (domain 4). Personal connections are highly important to ATSI peoples [61, 62] and having large extended families and community networks readily available provides resources, a sense of belonging and reinforces cultural identity [62, 63]. Culture and identity are central to Aboriginal Australians’ perceptions of health and ill-health [16]. These perceptions relate to the social, emotional, and cultural well-being of the community – not just the physical well-being [63]. While perceptions of identity may vary between urban and remote Aboriginal Australian communities, core values and principles are consistent between Indigenous groups [64]. It is difficult to infer why the *Social Support for health* domain was not higher; possibly colonisation and previous forced separation and assimilation might be eroding the sense of social support. We are conducting further research using qualitative methods to explore this domain in more depth.

### Limitations

There are several limitations of this study. The HLQ has not been used in an ATSI population before, and the items may not represent this populations’ world view of health. Due to the cross-sectional design of this study, relationships should be interpreted as associations rather than causal. Feedback from the Aboriginal Health Workers, who read aloud each item to avoid stigmatising those with limited or no functional literacy ability, identified that the word ‘ill’ (question 5) and the phrase ‘different sources’ (questions 4 and 12) of the HLQ to be words/phrases not used often within this population. English is frequently a second language for ATSI peoples living in Mount Isa and from a cultural perspective, English words may not be transferrable or appropriate. Cultural beliefs and world-views are important factors in health decisions and although Hawkins et al., [48] provides some evidence that the HLQ items and constructs are understood as intended, their study did not include ATSI participants. Further research of the validity and reliability of the HLQ in this population is needed. In addition, as the study was conducted with ATSI people living in one remote area of Australia and some people may have declined to participate due to their social or health professional relationships with the two Aboriginal Health Workers, the results may not be generalisable. Reporting bias may have also occurred with participants overstating their health literacy abilities to minimise possible embarrassment or shame as the two Aboriginal Health Workers live and work in the community. A response rate was not captured. Despite these limitations, the results indicate that health literacy abilities are lower than other Australian populations [43, 48, 53]. Lastly, we excluded those with a cancer diagnosis (which can be defined as a chronic disease), necessitating further health literacy research in the ATSI population.

## Conclusion

This is the first study to describe the chronic disease health literacy of ATSI people in Australia.

Health literacy abilities reflect the complexity of health information given to consumers and the healthcare system itself which is being navigated [65], thus predictors of health literacy in this population was an important discovery. This study found that age (< 55), gender (female), having one chronic disease, or having higher levels of education were associated with higher levels of health literacy across multiple HLQ domains. Despite the “Close the Gap” Australian government initiative over the last 12 yrs, inequities in health outcomes remain whereby ATSI peoples have lower life expectancy and higher rates of chronic disease than non-Indigenous Australians. Health literacy is critical to empowerment through improving people’s access and capacity to use health information. It is also contextual and there are challenges associated with social disadvantage along with multiple chronic diseases in this population. Our findings can inform local healthcare organisations to reform service delivery models and embed health literacy principles into routine clinical care that may assist with reducing health disparities for ATSI peoples.

## Additional files

Additional file 1: Demographic and health data questionnaire. (DOCX 385 kb)

Additional file 2: Characteristics of higher levels of health literacy. (DOCX 22 kb)

Additional file 3: Bivariate Mean scores and Effect Size for Health Literacy Questionnaire domains across demographic and health characteristics. (DOCX 34 kb)

## Data Availability

The datasets used and/or analysed during the current study are available from the first author on reasonable request.

## Abbreviations

AMC: Aboriginal community controlled medical centre
ANOVA: Analysis of variance
ATSI: Aboriginal and/or Torres Strait Islander
CI: Confidence Interval
ES: Effect size
HLQ: Health Literacy Questionnaire
HREC: Human Research Ethics Committee
IQR: Interquartile range
QUT: Queensland University of Technology
SD: Standard deviation
TAFE: Technical and Further Education
WHO: World Health Organization
