# Correction to: Health literacy in indigenous people with chronic disease living in remote Australia

**DOI:** 10.1186/s12913-019-4411-8

**Published:** 2019-08-14

**Authors:** Haunnah Rheault, Fiona Coyer, Lee Jones, Ann Bonner

**Affiliations:** 1 School of Nursing, Queensland University of Technology, Brisbane, Australia; 2 Advanced Heart Failure and Transplant Unit, The Prince Charles Hospital, Rode Road, Chermside, Brisbane, QLD 4032 Australia; 3 Intensive Care Services, Royal Brisbane and Women’s Hospital, Brisbane, Australia; 4 Institute of Health and Biomedical Innovation, Queensland University of Technology, Brisbane, Australia; 5 Kidney Health Service, Metro North Hospital and Health Service, Brisbane, Australia; 6 NHMRC Chronic Kidney Disease Centre of Research Excellence, University of Queensland, Brisbane, Australia

**Correction to: BMC Health Serv Res**

**https://doi.org/10.1186/s12913-019-4335-3**

In the original publication of this article [1], some values are missing in Table 3. Table 3 is revised in the updated figure below:

**Table 3:** Health Literacy Questionnaire scores (*n* = 200)

HLQ domain	Mean (SD) [95% CI]
	Range 1 (lowest) – 4 (highest)
1. Healthcare provider support	2.76 (0.52) [2.69, 2.83]
2. Having sufficient health information	2.59 (0.52) [2.51, 2.66]
3. Actively managing health	2.64 (0.49) [2.57, 2.70]
4. Social support for health	2.84 (0.52) [2.76, 2.91]
5. Critical appraisal	2.41 (0.55) [2.33, 2.49]
	Range 1 (lowest) – 5 (highest)
6. Active engagement with healthcare providers	3.14 (0.72) [3.01, 3.24]
7. Navigating the healthcare system	3.13 (0.75) [3.01, 3.23]
8. Ability to find good health information	2.89 (0.73) [2.79, 2.99]
9. Reading and understanding health information	2.82 (0.78) [2.72, 2.93]

*Abbreviations: HLQ* Health Literacy Questionnaire, *SD* standard deviation, *CI* confidence interval

The original article has been corrected.
