# Supplementary material for: Health literacy in Indigenous people with chronic disease living in remote Australia
Source: BMC Health Serv Res. 2019 Jul 26;19:523. doi: 10.1186/s12913-019-4335-3 (PMC6659262; doi:10.1186/s12913-019-4335-3)
Supplement: Supplementary file 1 — Demographic and health data questionnaire. (DOCX 385 kb) [file 12913_2019_4335_MOESM1_ESM.docx]

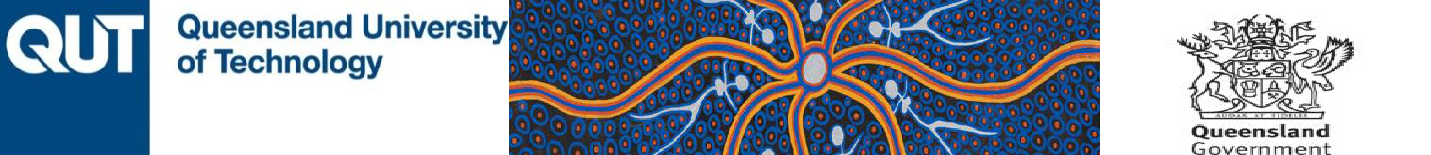


**Health literacy research in chronic disease**

**1. Please circle**
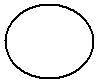
 **who is filling out this form?**

| You | | Family/friend |  | Researcher |  | Other__________________ | | | |  |
| --- | --- | --- | --- | --- | --- | --- | --- | --- | --- | --- |
| **2. How old are you? ________** years | | | |  |  |  |  |  |  |  |
| **3. Please circle** | | **your gender?** | | Male | Female | |  |  |  |  |
| **4. Please** √ **which chronic disease you have, and circle** | | | | | | **how long you have had this chronic** | | | |  |
| **disease. There can be more than one answer** | | | | |  |  |  |  |  |  |
|  |  |  | |  | | | | |  |  |
|  | **Chronic** | **Yes No** | | **How long have you had your chronic disease?** | | | | | |  |
|  | **disease** | **✓** | **X** | **Please Circle** |  |  |  |  |  |  |
|  |  |  |  |  |  |  |  |  |  |  |
|  |  |  |  |  |  |  |  |  |  |  |
|  |  |  |  |  |  |  |  |  |  |  |
|  | **Heart** |  |  | Less than 1 | 1-3 | 3-5 | 5-10 | More than | |  |
|  | **(cardiac)** | |  | Year | years | years | years | 10 years | |  |
|  |  |  |  |  |  |  |  |  |  |  |
|  | **Lungs** |  |  | Less than 1 | 1-3 | 3-5 | 5-10 | More than | |  |
|  | **(respiratory)** | |  | Year | years | years | years | 10 years | |  |
|  |  |  |  |  |  |  |  |  |  |  |
|  | **Kidney** |  |  | Less than 1 | 1-3 | 3-5 | 5-10 | More than | |  |
|  | **(renal)** |  |  | Year | years | years | years | 10 years | |  |
|  |  | |  |  |  |  |  |  |  |  |
|  | **Diabetes** | |  | Less than 1 | 1-3 | 3-5 | 5-10 | More than | |  |
|  |  |  |  | year | years | years | years | 10 years | |  |
|  |  |  |  |  |  |  |  |  |  |  |
|  | **Other** |  |  | Less than 1 | 1-3 | 3-5 | 5-10 | More than | |  |
|  | **(please list):** | |  | year | years | years | years | 10 years | |  |


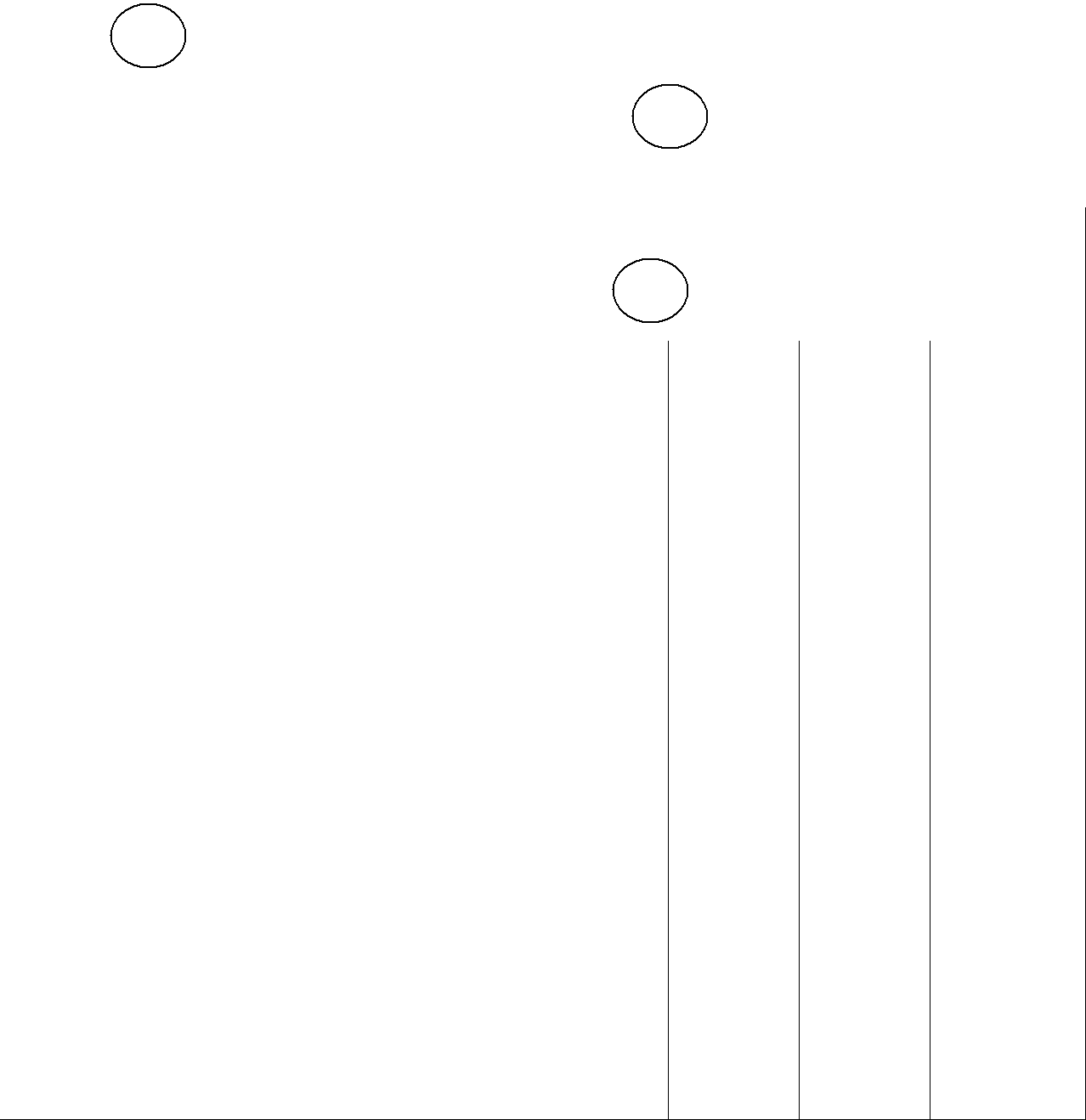


| **Other** | Less than 1 | 1-3 | 3-5 | 5-10 | More than |
| --- | --- | --- | --- | --- | --- |
| **(please list):** | year | years | years | years | 10 years |

1. **Please write the total number of medications you take every day (including tablets, insulin, puffers, patches) __________________________________________________________________________**
2. **Please make a** √ **on what condition you take your medications for. There can be more than one answer.**

|  | **Yes** | **No** |  | **Yes** | **No** |  |
| --- | --- | --- | --- | --- | --- | --- |
|  | **✓** | **X** |  | **✓** | **X** |  |
|  |  |  |  |  |  |  |
|  |  |  |  |  |  |  |
| Blood pressure |  |  | Pain |  |  |  |
|  |  |  |  |  |  |  |
| Chest pain |  |  | Lower heart beat |  |  |  |
|  |  |  | (beta-blockers) |  |  |  |
|  |  |  |  |  |  |  |
| Fluid |  |  | Puffers (lung) |  |  |  |
|  |  |  |  |  |  |  |
| Diabetes |  |  | Blood thinner |  |  |  |
|  |  |  |  |  |  |  |
| Cholesterol |  |  | Vitamins |  |  |  |
|  |  |  |  |  |  |  |
| Other: |  |  | Other: |  |  |  |
|  |  |  |  |  |  |  |

**7. Tick all of these health services you have used *the most* in the last 4 weeks. Why did you need to go?**

**Yes** **No** **Why did I go? Please write your reason down**

- **_X_**

Gidgee Healing

Ambulance service

Mt Isa ED department

Specialist DOCTOR

clinic Outpatients

Department

Specialist NURSE

clinic Outpatients

Department

Other GP service

RFDS clinics

1. **How many people do you live with?** ___________________________________


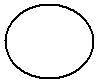


| **9. Please circle** | **the highest level of school you have completed** | | | | |  |
| --- | --- | --- | --- | --- | --- | --- |
|  |  |  |  | |  |  |
| Primary school | Year 7-9 | | Year 10 | | Year 11 | Year 12 |
| TAFE/Trade | University | |  | Prefer not to say | |  |
| **10. Please circle** | **how much money (total) your house earns in a year** | | | | |  |
| Less than $15,000 |  | Less than $20,000 | |  | $20,000-$30,000 |  |
| $30’000-$40,000 |  | More than $40,000 | |  | Prefer not to say |  |


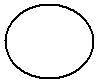


Thank you very much for your time. Your input is very valued and appreciated.
