# Supplementary material for: Health literacy in Indigenous people with chronic disease living in remote Australia
Source: BMC Health Serv Res. 2019 Jul 26;19:523. doi: 10.1186/s12913-019-4335-3 (PMC6659262; doi:10.1186/s12913-019-4335-3)
Supplement: Supplementary file 2 — Characteristics of higher levels of health literacy. (DOCX 22 kb) [file 12913_2019_4335_MOESM2_ESM.docx]

**Additional File 2.
Characteristics of higher levels of health literacy**

| **HLQ domain** | **Age:**  **< 55years**  Mean (95% CI) | **Gender:**  **Female**  Mean (95% CI) | **Number of Chronic  Diseases: 1**  Mean (95% CI) | **Income:  <$30,000/year**  Mean (95% CI) | | **Education:**  **TAFE/University/ Trade**  Mean (95% CI) | **Aboriginal Medical Health Clinic review past 4 weeks**  Mean (95% CI) |
| --- | --- | --- | --- | --- | --- | --- | --- |
| Range 1 ‘strongly disagree’ – 4 ‘strongly agree’ | | | | | | | |
| 1.Healthcare provider support |  |  |  |  | | 2.98(2.77, 3.18)****** |  |
| 2.Having sufficient health information | 2.73(2.62, 2.83)****** |  |  | 2.76(2.59, 2.94)***** | | 2.80(2.59, 3.00)***** |  |
| 3.Actively managing health | 2.72(2.63, 2.82)***** |  |  | |  |  |  |
| 4.Social support for health | 2.93(2.83, 3.03)***** |  |  | |  |  |  |
| 5.Critical appraisal | 2.59(2.49, 2.70)****** | 2.50(2.39, 2.62)***** | 2.59(2.47, 2.72)***** | |  |  |  |
| Range 1 ‘always difficult’ – 5 ‘always easy’ | | | | | | | |
| 6.Active engagement with healthcare providers | 3.38(3.25, 3.52)****** |  | 3.46(3.31, 3.54)****** | |  |  |  |
| 7.Navigating the healthcare system | 3.34(3.19, 3.48)****** | 3.24(3.09, 3.39)* | 3.37(3.21, 3.54)***** | |  |  |  |
| 8.Ability to find good health information | 3.08(2.95, 3.22)***** |  | 3.15(2.99, 3.31)****** | |  |  |  |
| 9.Reading and understanding health information | 3.05(2.89, 3.19)***** | 2.93(2.77, 3.09)***** | 3.15(2.98, 3.12)****** | |  |  |  |

*****p<0.05 ******p<0.01

Referent groups: Age: >55, Gender: Male, Number of chronic diseases: ≥2, Household income per annum: Prefer not to say, Education: Primary school, Attendance to a medical appointment at the local Aboriginal community controlled medical centre in the past 4 weeks (yes, no).
