# Supplementary material for: Health literacy in Indigenous people with chronic disease living in remote Australia
Source: BMC Health Serv Res. 2019 Jul 26;19:523. doi: 10.1186/s12913-019-4335-3 (PMC6659262; doi:10.1186/s12913-019-4335-3)
Supplement: Supplementary file 3 — Bivariate Mean scores and Effect Size for Health Literacy Questionnaire domains across demographic and health characteristics. (DOCX 34 kb) [file 12913_2019_4335_MOESM3_ESM.docx]

**Additional File 3**Bivariate Mean scores and Effect Size for Health Literacy Questionnaire domains across demographic and health characteristics.

| **Health Literacy Questionnaire domains** | | | | | | | | | |
| --- | --- | --- | --- | --- | --- | --- | --- | --- | --- |
|  | 1.Healthcare provider support  Mean (SD),  [95% CI] | 2.Having sufficient health information  Mean (SD),  [95% CI] | 3.Actively managing health  Mean (SD),  [95% CI] | 4.Social support for health  Mean (SD),  [95% CI] | 5.Critical appraisal  Mean (SD),  [95% CI] | 6.Active engagement with healthcare providers  Mean (SD),  [95% CI] | 7.Navigating the healthcare system  Mean (SD),  [95% CI] | 8.Ability to find good health information  Mean (SD),  [95% CI] | 9.Reading and understanding health information  Mean (SD),  [95% CI] |
| **Gender** | | | | | | | | | |
| *Male*  (Ref. category) | 2.72 (0.52)  [2.63, 2.82] | 2.53 (0.51)  [2.43, 2.63] | 2.60 (0.48)  [2.51, 2.69] | 2.81 (0.57)  [2.71, 2.91] | 2.34 (0.52)  [2.24, 2.45] | 3.08 (0.74)  [2.95, 3.22] | 3.03 (0.77) [2.88, 3.17] | 2.82 (0.71)  [2.68, 2.96] | 2.73 (0.77)  [2.58, 2.88] |
| *Female* | 2.80 (0.51)  [2.70, 2.91] | 2.66 (0.53)  [2.56, 2.77] | 2.68 (0.50)  [2.58, 2.79] | 2.88 (0.47)  [2.77, 2.98] | 2.50 (0.58)  [2.39, 2.62] | 3.21 (0.68)  [3.06, 3.36] | 3.24 (0.72) [3.09, 3.39] | 2.97 (0.75) [2.83, 3.13] | 2.93 (0.78)  [2.77, 3.09] |
| B (95% CI):  Female vs. Ref.  *Effect Size* | 0.08  (-0.06, 0.22)  0.15 | 0.13  (-0.01, 0.27)  0.53 | 0.08  (-0.05, 0.22)  0.16 | 0.07  (-0.07, 0.22)  0.13 | 0.16  (0.07, 0.31)  0.29 | 0.13  (-0.07, 0.33)  0.18 | 0.21  (0.06, 0.42)  0.28 | 0.15  (-0.05, 0.36)  0.20 | 0.20  (-0.02, 0.41)  0.26 |
| ANOVA | *p*=0.27 | *p*=0.07 | *p*=0.23 | *p*=0.33 | *p*=0.04 | *p*=0.21 | *p*=0.04 | *p*=0.13 | *p*=0.07 |
| **Age groups** | | | | | | | | | |
| *<55 years*  (Ref. category) | 2.86 (0.45) [2.76, 2.96] | 2.73 (0.45) [2.63, 2.83] | 2.72 (0.42)  [2.63, 2.82] | 2.93 (0.44)  [2.83, 3.03] | 2.59 (0.46) [2.49, 2.70] | 3.38 (0.64) [3.25, 3.52] | 3.34 (0.65) [3.19, 3.48] | 3.08 (0.67) [2.95, 3.22] | 3.05 (0.75) [2.89, 3.19] |
| *>55 years* | 2.66 (0.55) [2.51, 2.76] | 2.45 (0.55) [2.35, 2.55] | 2.56 (0.55) [2.46, 2.65] | 2.74 (0.58) [2.64, 2.85] | 2.23 (0.58) [2.13, 2.33] | 2.90 (0.71) [2.77, 3.04] | 2.91 (0.80) [2.77, 3.06] | 2.69 (0.74) [2.56, 2.84] | 2.60 (0.76) [2.45, 2.75] |
| B (95% CI):  >55 vs. Ref.  *Effect Size* | -0.2  (-0.34, -0.06)  0.39 | -0.28  (-0.42, -0.13)  0.55 | -0.17  (-0.30, -0.03)  0.33 | -0.18  (-0.33, -0.04)  0.37 | -0.36  (-0.51, -0.22)  0.68 | -0.48  (-0.67, -0.29)  0.71 | -0.43  (-0.63, -0.22)  0.59 | -0.39  (-0.58, -0.19)  0.55 | -0.45  (-0.66, -0.24)  0.59 |
| ANOVA | *p*=0.05 | *p*=<0.01 | *p*=0.01 | *p*=0.01 | *p*=<0.01 | *p*=<0.01 | *p*=<0.01 | *p*=<0.01 | *p*=<0.01 |
| **Education** | | | | | | | | | |
| *Primary*  (Ref. category) | 2.53 (0.55) [2.37, 2.69] | 2.35 (0.54)  [2.19, 2.51] | 2.51 (0.58)  [2.19, 2.51] | 2.63 (0.56)  [2.46, 2.79] | 2.12 (0.60)  [1.95, 2.23] | 2.78 (0.77)  [2.56, 3.00] | 2.75 (0.86)  [2.52, 2.98] | 2.54 (0.71)  [2.32, 2.77] | 2.41 (0.71)  [2.17, 2.65] |
| *Secondary* | 2.77 (0.53) [2.66, 2.88] | 2.67 (0.51)  [2.56, 2.78] | 2.67 (0.47)  [2.56, 2.78] | 2.91 (0.52)  [2.80, 3.02] | 2.43 (0.50)  [2.32, 2.78] | 3.19 (0.68)  [3.03, 3.34] | 3.14 (0.67)  [2.98, 3.30] | 2.92 (0.71)  [2.76, 3.08] | 2.83 (0.79)  [2.73, 3.06] |
| *TAFE/ University/ Trade* | 2.98 (0.57) [2.77, 3.18] | 2.80 (0.57)  [2.59, 3.00] | 2.79 (0.55)  [2.59, 3.00] | 2.98 (0.57)  [2.77, 3.19] | 2.56 (0.71)  [2.35, 2.78] | 3.29 (0.78)  [3.00, 3.57] | 3.34 (0.82)  [3.05, 3.64] | 3.07 (0.76)  [2.78, 3.36] | 3.05 (0.83)  [2.74, 3.35] |
| *Prefer not to say* | 2.82 (0.37) [2.69, 2.96] | 2.55 (0.44)  [2.42, 2.69] | 2.63 (0.42)  [2.42, 2.69] | 2.81 (0.45)  [2.68, 2.96] | 2.54 (0.45)  [2.39, 2.68] | 3.27 (0.62)  [3.08, 3.46] | 3.27 (0.69)  [3.08,3.47] | 3.02 (0.70)  [2.83, 3.21] | 2.92 (0.72)  [2.72, 3.13] |
| B (95% CI)  Secondary vs. Ref.  *Effect Size* | 0.24  (0.04, 0.43)  0.44 | 0.32  (0.13, 0.52)  0.61 | 0.16  (-0.02, 0.35)  0.30 | 0.28  (0.08, 0.48)  0.52 | 0.32  (0.11, 0.52)  0.56 | 0.41  (0.14, 0.68)  0.56 | 0.38  (0.10, 0.67)  0.50 | 0.38  (0.11, 0.65)  0.53 | 0.48  (0.19, 0.77)  0.56 |
| B (95% CI):  *TAFE/ University/ Trade* vs. Ref.  *Effect Size* | 0.45  (0.19, 0.70)  0.80 | 0.45  (0.19, 0.71)  0.81 | 0.28  (0.03, 0.53)  0.49 | 0.35  (0.08, 0.62)  0.62 | 0.45  (0.17, 0.72)  0.67 | 0.51  (0.15, 0.87)  0.65 | 0.59  (0.21, 0.96)  0.70 | 0.53  (0.16, 0.89)  0.72 | 0.64  (0.25, 1.03)  0.83 |
| B (95% CI):  Prefer not to say vs. Ref.  *Effect Size* | 0.29  (0.08, 0.50)  0.80 | 0.20  (-0.01, 0.41)  0.41 | 0.12  (-0.08, 0.32)  0.24 | 0.18  (-0.03, 0.40)  0.35 | 0.42  (0.19, 0.64)  0.79 | 0.49  (0.20, 0.78)  0.70 | 0.52  (0.22, 0.83)  0.66 | 0.47  (0.18, 0.77)  0.68 | 0.51  (0.19, 0.83)  0.71 |
| ANOVA | *p*=0.004 | *p*=0.002 | *p*=0.14 | *p*=0.02 | *p*=0.001 | *p*=0.004 | *p*=0.003 | *p*=0.006 | *p*=0.002 |
| **Income** | | | | | | | | | |
| *<$30,000*  (Ref. category) | 2.94 (0.45)  [2.76, 3.11] | 2.76 (0.48)  [2.59, 2.94] | 2.74 (0.48)  [2.57, 2.91] | 2.96 (0.45)  [2.78, 3.14] | 2.63 (0.51)  [2.44, 2.82] | 3.42 (0.66)  [3.17, 3.66] | 3.36 (0.79)  [3.10, 3.61] | 3.15 (0.77)  [2.90, 3.39] | 3.05 (0.79)  [2.78, 3.32] |
| *$30,000-$40,000* | 2.71 (0.62)  [2.57, 2.84] | 2.63 (0.56)  [2.49, 2.76] | 2.62 (0.57)  [2.49, 2.75] | 2.80 (0.56)  [2.67, 2.94] | 2.41 (0.63)  [2.26, 2.55] | 3.10 (0.74)  [2.91, 3.27] | 3.11 (0.76)  [2.92, 3.31] | 2.86 (0.68)  [2.68, 3.05] | 2.85 (0.85)  [2.65, 3.05] |
| *>$40,000* | 2.74 (0.48)  [2.59, 2.87] | 2.61 (0.53)  [2.47, 2.75] | 2.66 (0.45)  [2.53, 2.80] | 2.88 (0.55)  [2.74, 3.02] | 2.37 (0.56)  [2.22, 2.52] | 3.12 (0.77)  [2.93, 3.31] | 3.09 (0.77)  [2.89, 3.30] | 2.85 (0.77) [2.65, 3.05] | 2.78 (0.78)  [2.58, 2.99] |
| *Prefer not to say* | 2.74 (0.43)  [2.60, 2.88] | 2.42 (0.46)  [2.28, 2.56] | 2.58 (0.44)  [2.44, 2.71] | 2.76 (0.50)  [2.62, 2.91] | 2.33 (0.46)  [2.18, 2.48] | 3.06 (0.65)  [2.86, 3.25] | 3.03 (0.71)  [2.82, 3.24] | 2.81 (0.70)  [2.61, 3.01] | 2.82 (0.78) [2.47, 2.89] |
| B (95% CI):  $30,000-$40,000 vs. Ref.  *Effect Size* | -0.22  (-0.44, -0.01)  0.42 | -0.35  (-0.35, 0.08)  0.25 | -0.12  (-0.33, 0.09)  0.23 | -0.16  (-0.38, 0.06)  0.31 | -0.22  (-0.45, 0.01)  0.38 | -0.33  (-0.63, -0.02)  0.45 | -0.24  (-0.56, 0.07)  0.32 | -0.28  (-0.59, 0.03)  0.40 | -0.19  (-0.53, 0.13)  0.24 |
| B (95% CI):  >$40,000 vs. Ref.  *Effect Size* | -0.19 (-(0.42, 0.02)  0.43 | -0.15  (-0.37, 0.07)  0.29 | -0.07  (-0.28, 0.14)  0.17 | -0.08  (-0.31, 0.14)  0.16 | -0.25  (-0.49, -0.02)  0.48 | -0.29  (-0.61, 0.01)  0.42 | -0.26  (-0.58, 0.06)  0.35 | -0.29  (-0.61, 0.02)  0.39 | -0.26  (-0.60, 0.07)  0.34 |
| B (95% CI):  Prefer not to say vs. Ref.  *Effect Size* | -0.18  (-0.41, 0.03)  0.45 | -0.34  (-.56, -0.12)  0.72 | -0.16  (-0.38, 0.05)  0.38 | -0.20  (-0.43, 0.02)  0.42 | -0.29  (-0.53, -0.05)  0.62 | -0.36  (-0.67, -0.05)  0.55 | -0.32  (-0.65, 0.00)  0.44 | -0.34  (-0.65, -0.02)  0.46 | -0.37  (-0.71, -0.03)  0.30 |
| ANOVA | *p*=0.20 | *p*=0.02 | *p*=0.48 | *p*=0.30 | *p*=0.09 | *p*=0.11 | *p*=0.26 | *p*=0.17 | *p*=0.19 |
| **Number chronic disease/s** | | | | | | | | | |
| *1* (Ref. category) | 2.87 (0.49) [2.76, 2.99] | 2.75 (0.47) [2.63, 2.86] | 2.73 (0.46) [2.62, 2.84] | 2.94 (0.48) [2.82, 3.06] | 2.59 (0.51) [2.47, 2.72] | 3.46 (0.62) [3.31, 3.62] | 3.37 (0.63) [3.21, 3.54] | 3.15 (0.58) [2.99, 3.31] | 3.15 (0.70) [2.98, 3.12] |
| *≥2* | 2.69 (0.51) [2.60, 2.78] | 2.49 (0.53) [2.41, 2.58] | 2.59 (0.51) [2.53, 2.67] | 2.78 (0.54) [2.68, 2.87] | 2.31 (0.55) [2.21, 2.40] | 2.95 (0.71) [2.83, 3.07] | 2.98 (0.79) [2.85, 3.11] | 2.73 (0.77) [2.61, 2.86] | 2.63 (0.77) [2.49, 2.76] |
| B (95% CI):  ≥2 vs. Ref.  *Effect Size* | -0.18  (-0.33, -0.03)  0.36 | -0.25  (-0.39, -0.11)  0.52 | -0.14  (-0.28, 0.00)  0.28 | -0.16  (-0.31, -0.01)  0.31 | -0.30  (-0.45, -0.14)  0.53 | -0.51  (-0.71, -0.32)  0.76 | -0.39  (-0.60, -0.18)  0.54 | -0.42  (-0.62, -0.21)  0.62 | -0.52  (-0.74, -0.31)  0.71 |
| ANOVA | *p*=0.02 | *p*=0.01 | *p*=0.05 | *p*=0.038 | *p*=<0.01 | *p*=<0.01 | *p*=<0.01 | *p*=<0.01 | *p*=<0.01 |
| **Aboriginal community controlled medical centre review within the preceding four-weeks** | | | | | | | | | |
| *Yes* (Ref. category) | 2.76 (0.52) [2.68, 2.85] | 2.57 (0.52) [2.48, 2.66] | 2.64 (0.49) [2.56, 2.72] | 2.80 (0.51) [2.71, 2.88] | 2.43 (0.53) [2.34, 2.52] | 3.13 (0.72) [3.02 3.25] | 3.14 (0.73) [3.01, 3.26] | 2.89 (0.72) [2.77, 3.02] | 2.81 (0.77) [2.68, 2.94] |
| *No* | 2.76 (0.50) [2.62, 2.89] | 2.65 (0.54) [2.51, 2.78] | 2.64 (0.48) [2.52, 2.77] | 2.94 (0.56) [2.80, 3.07] | 2.38 (0.62) [2.34, 2.53] | 3.17 (0.73) [2.98, 3.36] | 3.10 (0.83) [2.91, 3.30] | 2.88 (0.77) [2.69, 3.07] | 2.87 (0.81) [2.66, 3.07] |
| B (95% CI):  No vs. Ref.  *Effect Size* | -0.01  (-0.16, 0.15)  0.00 | 0.07  (-0.08, 0.23)  0.16 | 0.01  (-0.14, 0.16)  0.00 | 0.14  (-0.20, 0.30)  0.26 | -0.05  (-0.22, 0.12)  0.08 | 0.03  (-0.18, 0.25)  0.05 | -0.04  (-0.027, 0.19)  0.05 | -0.01  (-0.24, 0.21)  0.01 | 0.06  (-0.18, 3.02)  0.01 |
| ANOVA | *p*=0.93 | *p*=0.36 | *p*=0.92 | *p*=0.08 | *p*=0.58 | *p*=0.76 | *p*=0.75 | *p*=0.91 | *p*=0.62 |

B denotes Mean difference with 95% confidence intervals. Small Effect Size = 0.2-0.5; Medium Effect Size = 0.5-0.8 and Large Effect Size = >0.8 (Cohen, 1988).

Abbreviations: TAFE= Technical and Further Education; Ref = Reference group.
